# Gut bacteria are essential for normal cuticle development in herbivorous turtle ants

**DOI:** 10.1038/s41467-021-21065-y

**Published:** 2021-01-29

**Authors:** Christophe Duplais, Vincent Sarou-Kanian, Dominique Massiot, Alia Hassan, Barbara Perrone, Yannick Estevez, John T. Wertz, Estelle Martineau, Jonathan Farjon, Patrick Giraudeau, Corrie S. Moreau

**Affiliations:** 1 CNRS UMR8172 EcoFoG, AgroParisTech, Cirad, INRAE, Université des Antilles, Université de Guyane, Kourou, France; 2 Université d’Orléans, CEMHTI CNRS UPR3079, Orléans, France; 3 Bruker Switzerland AG, Fällanden, Switzerland; 4 Department of Biology, Calvin University, Grand Rapids, MI USA; 5 Université de Nantes, CNRS, CEISAM UMR 6230, Nantes, France; 6 SpectroMaitrise, CAPACITÉS SAS, Nantes, France; 7 Department of Entomology and Department of Ecology and Evolutionary Biology, Cornell University, Ithaca, NY USA

**Keywords:** Evolution, Bacteria, Analytical chemistry, Biosynthesis

## Abstract

Across the evolutionary history of insects, the shift from nitrogen-rich carnivore/omnivore diets to nitrogen-poor herbivorous diets was made possible through symbiosis with microbes. The herbivorous turtle ants *Cephalotes* possess a conserved gut microbiome which enriches the nutrient composition by recycling nitrogen-rich metabolic waste to increase the production of amino acids. This enrichment is assumed to benefit the host, but we do not know to what extent. To gain insights into nitrogen assimilation in the ant cuticle we use gut bacterial manipulation, ^15^N isotopic enrichment, isotope-ratio mass spectrometry, and ^15^N nuclear magnetic resonance spectroscopy to demonstrate that gut bacteria contribute to the formation of proteins, catecholamine cross-linkers, and chitin in the cuticle. This study identifies the cuticular components which are nitrogen-enriched by gut bacteria, highlighting the role of symbionts in insect evolution, and provides a framework for understanding the nitrogen flow from nutrients through bacteria into the insect cuticle.

## Abstract (web summary)

Microbial symbionts can help their hosts metabolise diverse diets. A study on herbivorous turtle ants identifies the cuticular components which are nitrogen-enriched by gut bacteria, highlighting the role of symbionts in insect evolution.

## Introduction

Advanced genomic technologies and bioinformatics approaches have increased exponentially the diversity of known microbes in recent years. Nevertheless, understanding the role of microbial symbionts in hosts remains elusive. One approach for studying symbiosis is to manipulate microbes and monitor the consecutive changes within the host. This strategy has been applied successfully in the field of insect–microbe interactions demonstrating that beneficial bacteria increase the fitness^1,2^, tolerance to abiotic stressors^3^, nutrient assimilation^3,4^ chemical defense^5,6^, and cuticle formation^7,8^ of hosts. Although a limited number of biochemical processes by which microbes contribute to host’s metabolism are known, new molecular pathways are likely to be discovered.

The evolution of ants (∼150 million years)^9^ has been shaped by mutualistic interactions with microbes, which have permitted ants to radiate into new ecological niches^10^ becoming one of the most ecologically important insect groups^11^. The shift from nitrogen-rich carnivore/omnivore diets to nitrogen-poor herbivorous diets has evolved multiple times during ant evolution^12^. For example, the intracellular symbiont *Blochmannia*, associated with *Camponotus* ants produces several amino acids that help ants assimilate nitrogen—consequently improving larval development, fitness, and reproductive success^13,14^. Another example is the herbivorous turtle ants of the genus *Cephalotes* that that have low nitrogen diets and benefit from the nutritional contribution of gut microbes^10^. Genomic analysis of gut bacteria from turtle ant host species revealed the occurrence of bacterial genes related to nitrogen recycling and assimilation for the biosynthesis of essential and non-essential amino acids^15^. The microbially derived amino acids are taken up by the host, which presumably benefits them and supports a low nitrogen herbivorous diet. Interestingly, the conserved and phylogenetically distant gut symbionts of *Cephalotes* ants (Burkholderiales, Opitutales, Pseudomonadales, Rhizobiales, and Xanthomonadales) possess similar and complementary biosynthetic genes to recycle urea to produce multiple amino acids, suggesting, among other things, a high amino acid demand by the host.

It has been suggested that the amino acid tyrosine (Tyr) produced in significant quantities by bacteria during insect development is involved in cuticle formation^7^. This is supported by previous work in insect cuticle biochemistry showing the precursor role of aromatic amino acids in the metabolic pathways of melanization^16^ and sclerotization^17,18^. In beetles, several studies have shown that different host species have vertically transmitted endosymbionts, which contribute to the host’s cuticle formation, and in the absence of associated bacteria reported significantly thinner, softer, and pale/reddish cuticles instead of dark and thick cuticles^7,8,19,20^. The color, roughness, thickness, permeability, and mechanical properties of the cuticle vary across insects and these traits have played a role in insect adaptation into new ecological niches^21^. However, there are still gaps in our understanding of which cuticular constituents depend on the symbiont’s contribution as these past studies have mostly only noted physical changes in the absence of symbionts without identifying any molecular components of the cuticle impacted by symbionts. Symbiont contribution to cuticle formation emphasizes the potential important impact of symbiosis in insect evolution. Therefore, based on the fact that *Cephalotes* cuticles are very tough and often dark, we hypothesize that gut bacteria participate in the cuticle formation of arboreal herbivorous turtle ants. Although the combination of ^15^N isotopic enrichment experiment with ^15^N solid-state NMR has been a successful methodology to reveal the molecular structure of the chitin–catecholamide–protein matrix, specifically for characterizing the nitrogen–carbon bonds in the protein–catecholamide and inter-catecholamide cross-links^22,23^, this approach has not been used in recent studies of insect cuticle and never to investigate if host-associated bacteria contribute to these processes.

In this work, we perform feeding experiments with urea-^15^N_2_ in the presence or absence of antibiotics to reveal the contribution of bacteria in cuticle formation during insect development. Our approach consists of using NMR to document and characterize the changes in the gut and cuticle including the ^15^N-enriched products in the chitin–catecholamide–protein matrix. Overall, we report the biomolecular mechanism of cuticle formation assisted by gut bacteria in herbivorous ants.

## Results and discussion

### Study species and experimental manipulation

In herbivorous *Cephalotes* turtle ants their nitrogen-poor diet is supplemented by amino acids produced by gut bacteria from recycling nitrogen host waste such as urea^15^. These bacteria almost exclusively belong to families Burkholderiales, Opitutales, Pseudomonadales, Rhizobiales, and Xanthomonadales, are highly related across *Cephalotes* species, and possess genes involved in nitrogen recycling and assimilation. Labeled urea-^15^N_2_ was added to the sterile diet of *Cephalotes varians* of six different colonies for a sufficient period of time to ensure the full development of individuals from larvae to adults and also treated half of the colonies with antibiotics to compare to untreated colonies. Despite the difficulty of rearing *Cephalotes* species in laboratory, five larvae from each group fully developed to the adult stage and individuals from independent colonies were pooled for analysis.

### Bacterial community, isotopic, and cuticle response across treatment

To assess if turtle ant gut bacteria were disrupted in our paired feeding experiments, we confirmed the bacterial communities had been knocked down through 16S rRNA qPCR and amplicon sequencing. Our antibiotic treatment reduced the bacterial quantity in our samples from 14,033 to 6607 16S rRNA copy numbers (Supplementary Table 1), and analysis of the bacterial community diversity in our antibiotic treatment samples found almost all transient and environmental contaminates compared to our intact bacterial communities, which were dominated by symbiotic bacterial amplicon sequence variants (Supplementary Fig. 1). To measure the contribution of gut bacteria to the cuticle, we have quantitative and qualitative evidence. The shift in *δ*^15^N values in the cuticle measured by isotope-ratio mass spectroscopy demonstrates an impressive contribution of bacteria with a fourfold increase in the untreated samples compare to sample from the antibiotics experiment (Supplementary Table 2). Scanning electronic microscopy reveals that the cuticle of untreated *C. varians* individual is twofold thicker compare to treated individual (Supplementary Fig. 2).

### Quantification of aromatic amino acids in turtle ant guts

For the NMR experiments of pooled gut extracts, a proton ^1^H spectrum was recorded in order to track the aromatic amino acids, which are the precursors of melanin and the chitin–protein catecholamine cross-linkers in the cuticle (Supplementary Fig. 3). Resonances of interest appeared at low intensity in the aromatic region (6.5–8.5 ppm). The recorded 2D ^1^H–^1^H correlation spectroscopy (COSY) map (Supplementary Fig. 4) shows a number of correlations between metabolites, specifically two major coupled spin systems in the aromatic area (6.9/7.2 and 7.3/7.4 ppm), which were tentatively identified as Tyr and phenylalanine (Phe) resonances (Supplementary Fig. 4). To improve the identification of aromatic amino acids, a zero-quantum filtered total COSY experiment was carried out (Supplementary Figs. S5 and S6). The correlations between aliphatic resonances H^α^ and H^β^ were detected for Phe and Tyr and the aromatic area of the 2D TOCSY spectra shows the Phe and Tyr intra-aromatic correlations as well as other less concentrated aromatic amino acids histidine (His) and tryptophan (Trp) (Supplementary Fig. 6). The diffusion order spectroscopy (DOSY) experiment unambiguously extracts the ^1^H NMR spectra of Phe and Tyr due to their close but different logD values (−9.44 for Tyr and −9.42 for Phe) (Supplementary Fig. 6), thus making it possible to quantify the respective concentration of Phe and Tyr in a single measurement of five pooled individuals of the gut extract samples from the antibiotic treated and untreated experiments (Supplementary Table 3). A similar concentration of Phe and Tyr at ~11 μM without antibiotic treatment was calculated and this value drops to twofold lower (~5 μM) in the gut extract of the antibiotic treated group.

### Identification of cuticular components impacted by gut bacteria

**Fig. 1:**
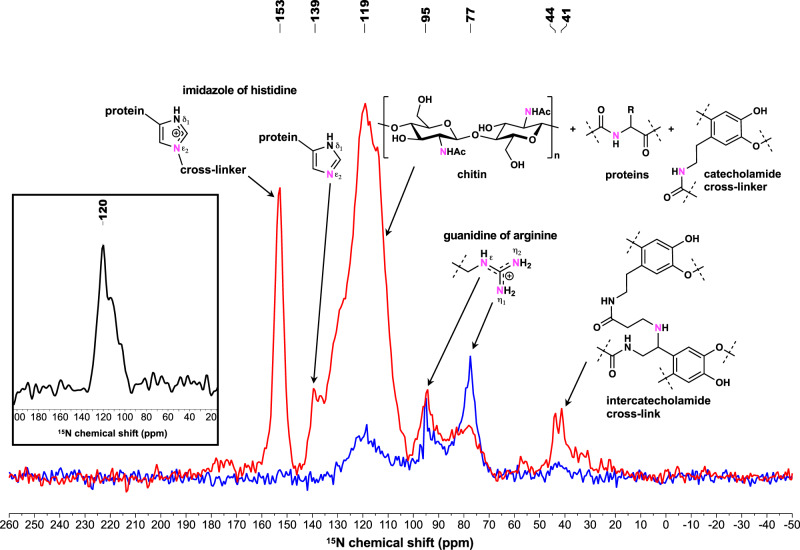
CP-MAS ^15^N NMR spectra of ant cuticle. Untreated (red line) and antibiotic treated (blue line) *C. varians* samples. Both spectra are normalized with respect to sample mass in the rotor and the number of accumulations. Enriched nitrogen is represented in pink. Inset depicts the ^15^N NMR spectrum of cuticle at natural isotopic abundance of unlabeled untreated *C. varians* ants.

**Fig. 2:**
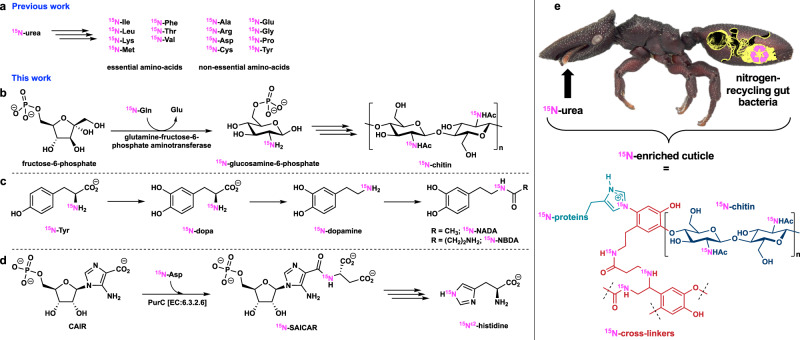
Gut bacteria contribution in nitrogen flux. **a** Biosynthetic pathways impacted by gut bacteria in the production of amino acids from previous work^15^ and molecular components of the cuticle inferred in this study (**b**–**d**). **e** Overall representation of gut bacteria contribution to ant cuticle (proteins, chitin, and cross-linkers are shown in different colors). Enriched nitrogen is represented in pink. Ant photograph used with permission from the Field Museum of Natural History (FMNHINS 0000 062 654).

The ^13^C cross-polarization magic-angle spinning (CP-MAS) NMR spectra of samples (antibiotics treated or untreated *C. varians* individuals) were recorded (Supplementary Fig. 7). Although resonances from chitin, proteins, and catecholamide were detected in agreement with previous work^22^, no significant difference was noticed between ^13^C resonances of antibiotic treated and untreated samples as expected as we did not enrich ^13^C in their diet. In contrast, the CP-MAS ^15^N NMR spectrum appeared greatly dissimilar (Fig. 1). The ^15^N resonances in antibiotic treated samples were qualitatively (number of peaks) and quantitatively (peak intensity) less compared to ^15^N resonances in the spectra of untreated samples, and different from the ^15^N NMR spectra at natural isotopic abundance of unlabeled untreated *C. varians* ants which is characterized by a single large resonance at 120 ppm (Fig. 1). Based on previous work on solid-state ^15^N NMR spectroscopy of insect cuticle^22,23^, we attempted to annotate all ^15^N resonances (Fig. 1 and Supplementary Table 4). The main ^15^N-enriched resonances in untreated samples, a broad peak at 100–145 ppm with a maximum at 119 ppm, represent the overlapping peaks of different ^15^N amide resonances in chitin (Supplementary Figs. S8 and S9), cuticular proteins, and catecholamide cross-linkers. We confirm the presence of ^15^N-enriched chitin in untreated *C. varians* individuals with a double cross-polarization {^1^H}–{^15^N}–^13^C experiment using a cryogenically cooled CP-MAS probe, which shows the resonances of the C1 and C2 carbons in close proximity to the ^15^N amide to allow polarization exchange of the ^15^N amide function in chitin (Supplementary Fig. 10). This suggests that the aminotransferase adds a glutamine amino acid ^15^N-enriched by gut bacteria to fructose-6-phosphate to form ^15^N-labeled chitin in *Cephalotes* cuticle (Fig. 2b)^24^. The broad peak (100–145 ppm) reflects the amide functional group in chitin overlapping with additional amide resonances of cuticular proteins composed of ^15^N-enriched amino acids and catecholamide cross-linkers *N*-acetyldopamine (NADA) and *N*-β-alanyldopamine (NBDA) from presumably microbially derived ^15^N-enriched Phe and Tyr precursors (Fig. 2c).

The second resonances of interest which was found in the untreated sample only is the peak at 153 ppm identified as the N-substituted nitrogen (N^ε2^) in the protonated imidazole ring of His (Fig. 1). The complexity of isotropic chemical shifts of the N^δ1^ and N^ε2^ sites in His has been thoroughly described for NMR spectroscopy of enzymes^25–27^. The chemical shift of 153 ppm was assigned to the N-substituted N^ε2^ site of protonated His covalently bonded to chitin, whereas the non-protonated N^ε2^ site of neutral His was putatively attributed to the resonance at 139 ppm. Neither of the resonances of the protonated and non-protonated N^δ1^ site in His were observed, respectively, at 144–147 and 225–230 ppm. The attributed N^ε2^ resonances of His only detected in untreated *C. varians* ants highlight an unexpected contribution of the gut bacteria to the nitrogen connecting cuticular proteins and chitin in the cuticle. Such enrichment has to occur early in His biosynthesis with a ^15^N-enriched aspartame as the source of ^15^N (Fig. 2d and Supplementary Fig. 11)^28^.

The ^15^N resonances between 70 and 100 ppm are attributed to the guanidine nitrogen (N^η1^, N^η2^, N^ε^) of arginine and the N^η^ resonance at 77 ppm has a quantitative higher enrichment in antibiotic treated samples. Enhanced resonance in the ^15^N spectra of untreated samples, between 40 and 50 ppm, represent the secondary amine resonance previously identified in the inter-catecholamide cross-links^23^. In insect cuticle, catecholamide cross-linkers NADA and NBDA connect chitin and proteins during sclerotization. It has been shown that another type of cross-link can occur from the reaction between NBDA and *N*-β-alanylnorepinephrine to form catecholamide–catecholamide cross-links^23^. These results underline that ^15^N-recycling by gut bacteria contribute to the formation of different types of cross-links in the cuticular chitin–protein matrix (Fig. 2e).

Originally, we expected ^15^N-enrichment to occur in the melanization and sclerotization pathways since the gut bacteria increase the concentration of Tyr and Phe, which are the biosynthetic precursors of melanin and catecholamide cross-linkers (Supplementary Fig. 12). The broad ^15^N NMR resonances of melanin, which is characterized by multiple resonance peaks (100–190 ppm) from a complex mixture of catechol- and dihydroxyindole-based oligomers^29,30^, were not identified in our samples, as confirmed by comparison with spectra of melanin standards (Supplementary Figs. 13 and 14). This is likely because the quantity of melanin in a ∼10 mg sample was too small to be detected. Even though the cuticle of antibiotic treated *C. varians* individuals appears to be as dark as untreated individuals it was not possible to assess the contribution of gut bacteria to melanin production.

### Implications for insect evolution

From an evolutionary standpoint, the functional redundancy related to the nitrogen flux in the genomes of the conversed symbionts (Burkholderiales, Opitutales, Pseudomonadales, Rhizobiales, and Xanthomonadales) in the gut of herbivorous turtle ants is intriguing. Maybe having different bacterial families, which can feed on various nutritional sources, allow for the constant production of amino acids for the host. Such multipartite mutualism is contrasting with the single strain *Nardonella* bacterial endosymbiont of several weevil beetle species, which contributes to cuticle formation^7^. *Nardonella*’s small genome (0.2 Mb) retains only the Tyr metabolic pathway and no other amino acid biosynthetic pathway was identified. Similar results were reported for the *Sitophilus* cereal weevil associated with the *Sodalis pierantonius* endosymbiont which provides Tyr and Phe during metamorphosis to contribute to cuticle formation^19^. Tyr and Phe produced by the symbiont are assumed to be involved in sclerotization and melanization processes as reddish and soft cuticles are formed in the absence of the symbiont. In comparison, we have demonstrated that turtle ant-associated bacteria not only contribute to the production of Tyr and Phe but to the formation of chitin, cuticular proteins, and catecholamine cross-linkers (Fig. 2e). Therefore the macromolecular structure, thickness, permeability, and mechanical properties of ant cuticle are likely impacted by gut bacteria suggesting that the conserved symbionts have indirectly contributed to the cuticle-associated traits, which have played a role in turtle ant adaptation into new ecological niches.

As we discover the roles played by mutualistic bacteria in hosts, the study of nitrogen flux is revealing which host physiological functions benefit from the microbial enrichment of amino acids. We report multiple lines of evidence that the nitrogen-recycling activity of gut bacteria is involved in several biosynthetic pathways contributing to host cuticle formation. We identify the contribution of gut bacteria at the molecular level to produce cuticular components, i.e., chitin, proteins, and cross-linkers. Overall these results demonstrate the important role of symbiotic bacteria in insect ecology and evolution.

## Methods

### Collection

*C. varians* colonies (*N* = 6) were collected in the Florida Keys, USA (Florida Department of Environmental Protection scientific research permit 04251635; US Fish and Wildlife research permit FFO4RFKD-2015-0). Voucher specimens are deposited in the Cornell University Insect Collection.

### Feeding experiment

For the feeding experiment with urea-^15^N_2_ (Sigma-Aldrich, ref: 316930), six colonies were split into two treatment groups. In the first treatment, the colony were subjected to antibiotic feeding to remove their gut bacteria through rearing on 30% (weight/volume) sucrose water containing 0.01% (w/v) of each Tetracycline, Rifampicin, and Kanamycin. Untreated colonies from the second treatment group consumed only 30% sucrose water. After 3 weeks of antibiotics treatment both treated and untreated colonies were reared upon the same 30% sucrose water diet also containing 1% (w/v) urea-^15^N_2_.

### Bacterial 16S rRNA qPCR and amplicon sequencing

The abdomen of one adult from each treatment group was extracted with the Qiagen Powersoil Kit (Qiagen, Germantown, MD) following the manufactures protocol. We amplified the bacterial region of 16S rRNA with primers described in Caporaso et al.^31^, following the Earth Microbiome Project protocol [515f primer (5′-GTGCCAGCMGCCGCGGTAA) and 806r primer (5′-GGACTACHVHHHTWTCTAAT); for details see: http://www.earthmicrobiome.org/emp-standard-protocols/16s/]. An Illumina MiSeq run using MiSeq V2 Reagent Kit 300 Cycles (150 × 150) was performed using the primers and procedures described previously^31^. We measured the quantity of bacterial DNA present with quantitative PCR of the bacterial 16S rRNA gene using the same primers as above. All qPCRs were performed in triplicate on a CFX Connect Real-Time System (Bio-Rad, Hercules, CA). Standard curves were created from serial dilutions of linearized plasmid containing inserts of the *E. coli* 16S rRNA gene and melt curves were used to confirm the absence of qPCR primer dimers. The resulting triplicate quantities were averaged before calculating the number of bacterial 16S rRNA gene copies per microliter of DNA extraction.

### Preparation of ant samples for chemical analysis

After 8 weeks of rearing the larvae that became adults over the course of feeding experiments were collected and individuals were pooled for each treatment group (treated *N* = 5, and untreated *N* = 5). The gut of these individuals (*N* = 5 per treatment) was dissected and metabolites were extracted by grinding pooled guts with a pestle in an Eppendorf tube (1.5 mL) containing a solvent mix (0.5 mL, ethyl acetate/methanol/water: 3:2:1). The mixture was centrifuged (2 min, 1677 × *g* rpm) and the supernatant was transferred to a vial prior to drying under vacuum. After dissecting guts and removing internal tissue, ant cuticles from the whole organisms were crushed in a mortar and washed two times successively with dichloromethane and methanol. The washed cuticle parts were dried at 50 °C for 3 h before further solid-state NMR experiments. For solution-state NMR, samples were composed of extracts dissolved in D_2_O and buffered at pH = 7.42 with 0.8 mM TSP-*d*_4_ as a chemical shift and concentration reference.

### Solution-state NMR spectroscopy

^1^H 1D and 2D NMR spectra were acquired on a Bruker Avance III HD spectrometer at 700.13 MHz, equipped with a ^1^H^/13^C/^15^N/^2^H cryoprobe at a low temperature: 277 K to prevent any degradation of the metabolites. For all 1D and 2D experiments, a continuous wave presaturation on the residual H_2_O was applied during the recycling time. Quantitative ^1^H 1D NMR spectra were recorded with a recycling time d1 of 25 s (as five times the highest T1 ^1^H), in quadriplicate with TSP-d_4_ as an internal reference. For the 2D DOSY experiment a stimulated echo with bipolar gradients including a longitudinal eddy current delay was chosen. A 6k × 56 matrix was acquired, zero-filled to 16k × 56 and apodized in F2 by a decreasing exponential with 2 HZ line broadening. A recycling time d1 = 5 s was used as well as a diffusion time d20 = 200 ms, with a gradient length of 900 and 200 ms of resting delay, a linear gradient ramp was used from 2 to 95% of the maximum gradient amplitude (50 G cm^−1^) and a number of accumulations NS = 200. An inverse Laplace transform was applied to obtain the 2D DOSY map displaying the diffusion coefficients along the indirect dimension. 2D COSY and ZQF-TOCSY spectra were also recorded. Further experimental parameters can be found in the figure captions. Tyr and Phe resonances were identified relying on the Biological Magnetic Resonance Bank database.

### Solid-state NMR spectroscopy

^15^N solid-state NMR experiments were carried out on a Bruker Avance III spectrometer operating at 9.4 T (Larmor frequency of 40.5 MHz) using a 4 mm magic-angle spinning (MAS) double resonance probe head. Around 10 mg of cuticle per treatment (five individuals) was packed in a 50 µL HRMAS rotor to ensure a proper centering of the sample. ^1^H–^15^N cross-polarization (CP-MAS) experiments were performed at a MAS frequency of 14,286 Hz with a contact time of 1 ms (^1^H and ^15^N radiofrequency fields during CP of ca. 55 and 40 kHz, respectively), and with a recycling delay of 1 s. The experimental time was about 2–2.5 days for each spectrum. The ^15^N spectra were referenced using L-tyrosine (*δ* = 40.4 ppm). The ^1^H–^15^N–^13^C double CP-MAS experiment was carried out on a Bruker Advance Neo spectrometer operating at 14.1 T (^13^C Larmor frequency of 150.9 MHz) using a 3.2 mm Bruker CP-MAS CryoProbe^TM^^32^. The MAS frequency was 11 kHz, and the ^1^H–^15^N and ^15^N–^13^C contact times were 1.5 and 7 ms, respectively. The recycle delay was 3.5 s, and the 65,536 scans were accumulated.

### Isotope-ratio mass spectrometry

Analysis was performed on a Thermo Delta V isotope-ratio mass spectrometer interfaced to a NC2500 elemental analyzer. In-house standards were calibrated against international reference materials provided by the International Atomic Energy Association. For the analytical sample run the overall standard deviation for the internal BSSL-100 standard (plant tissue) was 0.53‰ for *δ*^15^N and 0.05‰ for *δ*^13^C. Delta values obtained between the amplitudes of 100 and 10,000 mV for *δ*^15^N have an error associated with linearity of 0.65‰ and between 200 and 10,000 mV for *δ*^13^C error is 0.19‰. Isotope corrections are performed using a two-point normalization (linear regression) for all data using two additional internal standards (USGS40 and USGS41).

### Electronic microscopy

The gaster of one individual for each treatment group (treated and untreated) was removed from the body and cut along the dorsal midpoint of the second abdominal segment. Each gaster was mounted on double-sided carbon sticker attached to aluminum stubs. Secondary electron images were recorded on a FEI Quanta 250 ESEM operating between 10 and 15 kV and with a working distance of 10 mm. Images for each individual were taken in the same area of the cuticle.

### Reporting summary

Further information on research design is available in the Nature Research Reporting Summary linked to this article.

## Supplementary information

Supplementary Information

Reporting Summary

## Data Availability

Sequence data and NMR spectroscopic data are available, respectively, in the NCBI SRA database under the Accession Number PRJNA683914 and in the Dryad Digital Repository (https://datad ryad.org/) under 10.5061/dryad.d7wm37q0h.
