# Supplementary material for: Gut bacteria are essential for normal cuticle development in herbivorous turtle ants
Source: Nat Commun. 2021 Jan 29;12:676. doi: 10.1038/s41467-021-21065-y (PMC7846594; doi:10.1038/s41467-021-21065-y)
Supplement: Supplementary file 1 — Supplementary Information [file 41467_2021_21065_MOESM1_ESM.pdf]

## Supplementary information

# Gut bacteria are essential for normal cuticle development in herbivorous turtle ants

Christophe Duplais[1*], Vincent Sarou-Kanian[2], Dominique Massiot[2], Alia Hassan[3], Barbara Perrone[3], Yannick Estevez[1], John T. Wertz[4], Estelle Martineau[5,6], Jonathan Farjon[5], Patrick Giraudeau[5], Corrie S. Moreau[7*]

[1]CNRS UMR8172 EcoFoG, AgroParisTech, Cirad, INRAE, Université des Antilles, Université de Guyane, 1 avenue de France, 97310 Kourou, France
[2]Université d'Orléans, CEMHTI CNRS UPR3079, 1D avenue de la recherche scientifique, 45071 Orléans cedex 2, France
[3]Bruker Switzerland AG, Fällanden, Switzerland
[4]Calvin University, Department of Biology, Grand Rapids, MI 49546, USA
[5]Université de Nantes, CNRS, CEISAM UMR 6230, F-44000 Nantes, France
[6]SpectroMaitrise, CAPACITÉS SAS, 26 Bd Vincent Gâche, 44200 Nantes, France
[7]Cornell University, Department of Entomology and Department of Ecology and Evolutionary Biology, Ithaca, NY 14850, USA

Email: christophe.duplais@cnrs.fr or corrie.moreau@cornell.edu

## Table of contents

### Supplementary Figures

Supplementary Figure 1. Alpha diversity of the core bacterial ASVs in samples with 16S rRNA amplicon sequencing. ....3
Supplementary Figure. 2. Electronic microscopic transversal images and measurements of the cuticle (N=2). ....4
Supplementary Figure 3. Quantitative proton $^{1}$H NMR spectrum of treated pooled gut polar extracts (N=5). ....5
Supplementary Figure 4. $^{1}$H-$^{1}$H COSY of untreated pooled gut polar extracts (N=5). ....6
Supplementary Figure 5. 2D ZQF TOCSY of untreated pooled gut polar extracts (N=5). 7
Supplementary Figure 6. 2D ZQF TOCSY and 2D $^{1}$H DOSY spectra in the aromatic area. ....8
Supplementary Figure 7. CP-MAS $^{13}$C NMR spectra of antibiotic treated and untreated samples. ....9
Supplementary Figure 8. CP-MAS $^{15}$N NMR spectra of $\alpha$-chitine. ....10
Supplementary Figure 9. CP-MAS $^{15}$N NMR spectra of chitosan (85% purity). ....11
Supplementary Figure 10. Double CP-MAS $^{13}$C NMR spectra of the cuticle of $^{15}$N labelled untreated *C. varians* individuals. ....12
Supplementary Figure 11. Putative pathway of the enriched $^{15}N^{\varepsilon 2}$-histidine biosynthesis in ants. ....13
Supplementary Figure 12. Biosynthetic pathways of sclerotization and melanization. ....14
Supplementary Figure 13. CP-MAS $^{15}$N NMR spectra of melanin from *Sepia officinalis.* ....15
Supplementary Figure 14. CP-MAS $^{15}$N NMR spectra of synthetic melanin. ....16

### Supplementary Tables

Supplementary Table 1. Bacterial quantification (qPCR) of the 16S rRNA gene (515F / 806R). ....17
Supplementary Table 2. $\delta^{15}$N measurement in the cuticle by ICP-MS. ....18
Supplementary Table 3. 1H NMR quantification of aromatic amino acids. ....19
Supplementary Table 4. Chemical assignments of resonances in the CP-MAS $^{15}$N NMR spectra of *Cephalotes varians* cuticle. ....20

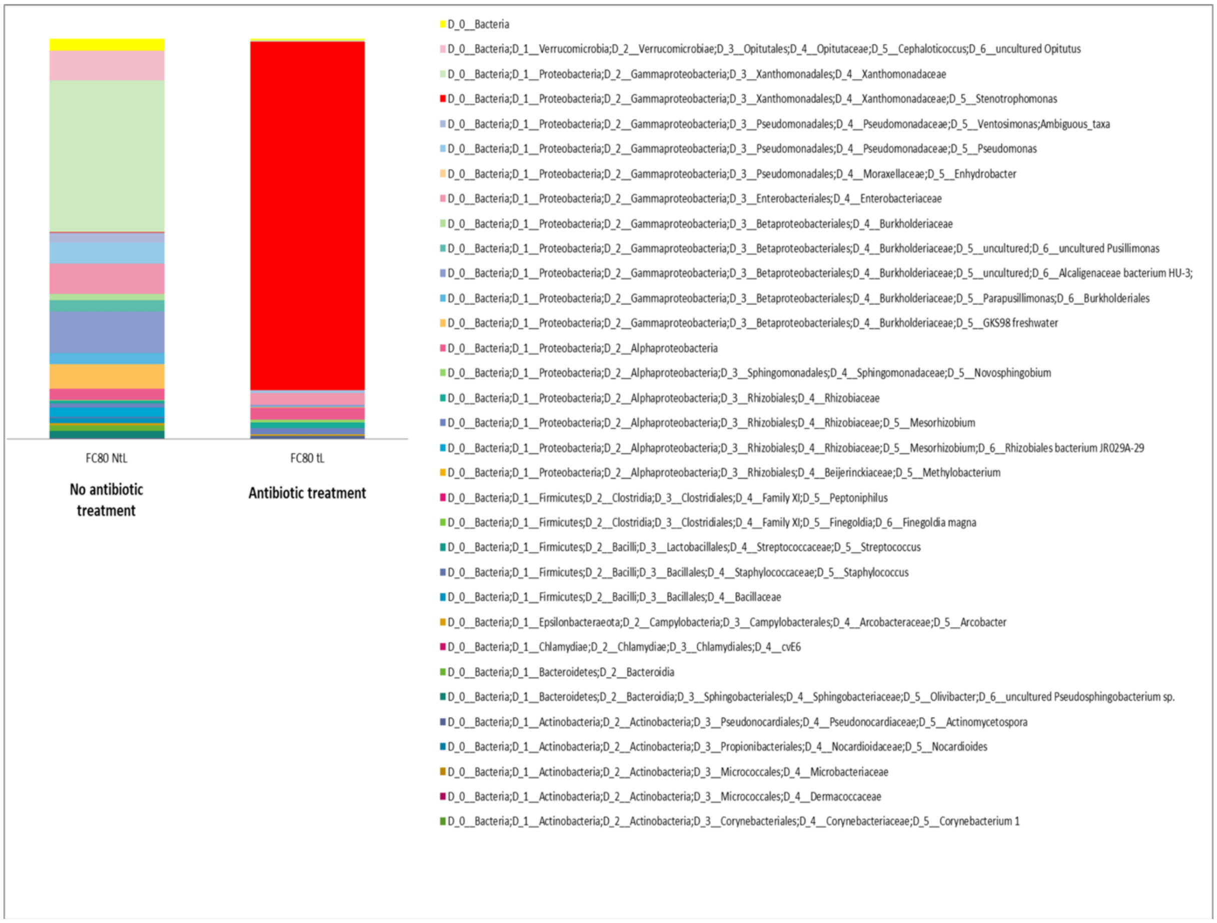


**Supplementary Figure 1. Alpha diversity of the core bacterial ASVs in samples with 16S rRNA amplicon sequencing.**

Bar graphs for each library (one column = community from a single sample) show the percentage of sequence reads classified to selected 99% similarity. Each color represents a distinct bacterium.

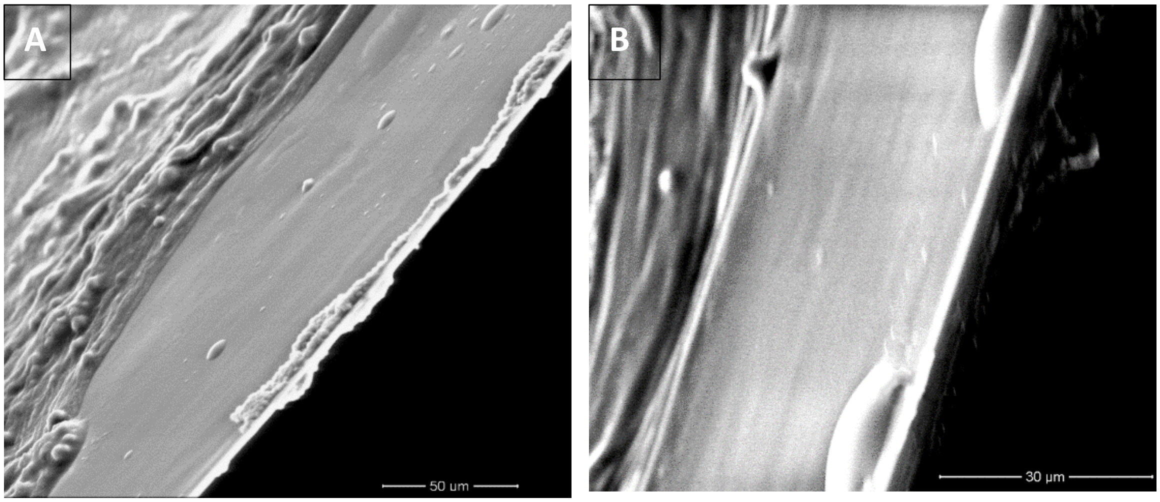


**Supplementary Figure. 2. Electronic microscopic transversal images and measurements of the cuticle (N=2).**

Untreated (A) and antibiotics treated (B) individuals measured in the same area. Each image was recorded with a Low Vacuum Secondary electron (LFD). Detector at (70 Pa) with a working distance of 10 mm. Scale bar in μm. Images not to scale.

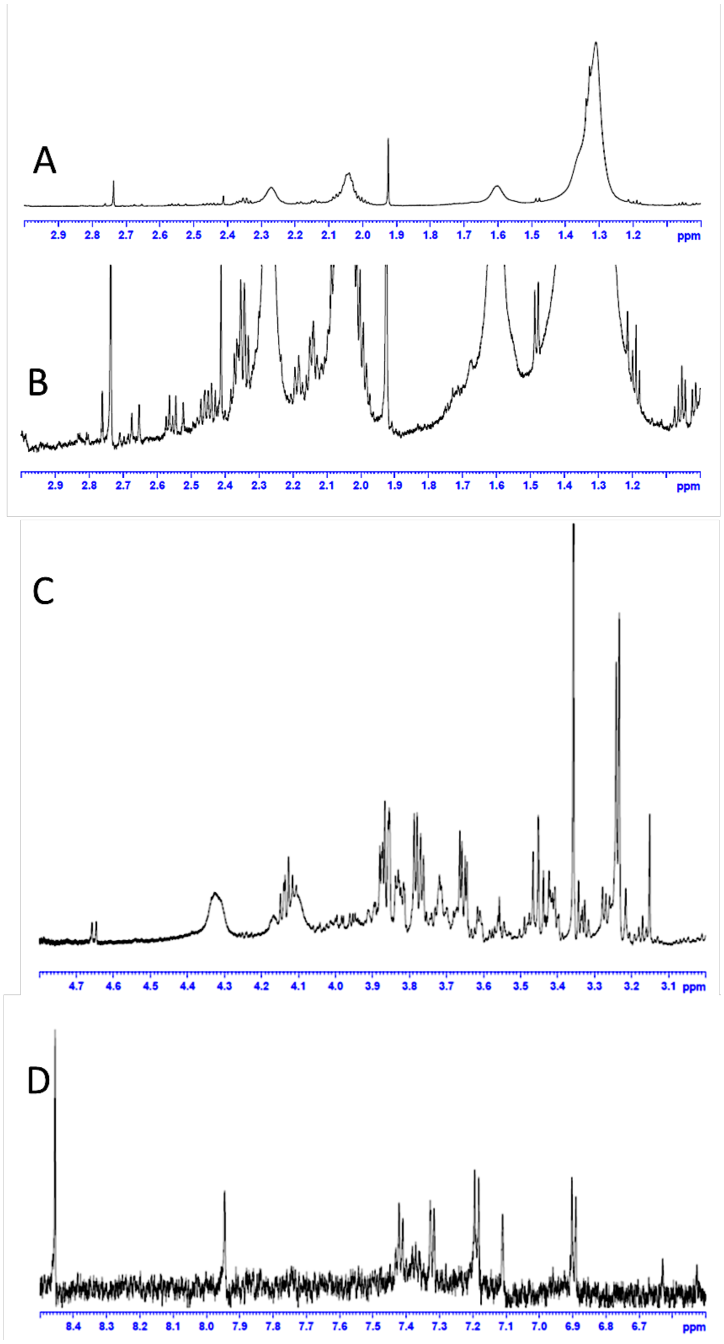


**Supplementary Figure 3. Quantitative proton $^{1}H$ NMR spectrum of treated pooled gut polar extracts (N=5).**

Proton spectrum between 0 and 3 ppm (A, B), 3 and 4.7 pm (C), and 6.5 and 8.5 ppm.

$^{1}H$ spectra (A, B, C: aliphatics and D: aromatics) with a presaturation applied on the resonance of the residual $H_2O$, recorded at 277K. The spectrum was recorded with 56 transients and a 25 s recycling delay. Free induction decays (FIDs) were acquired in 1.5 s with 25 k data points over a 12 pm spectral width. FIDs were zero-filled to 128 k and apodized with an exponential window function (lb = 2 Hz). The samples were buffered at pH=7.4 and TMSP-*d4* was used as a chemical shift and concentration reference.

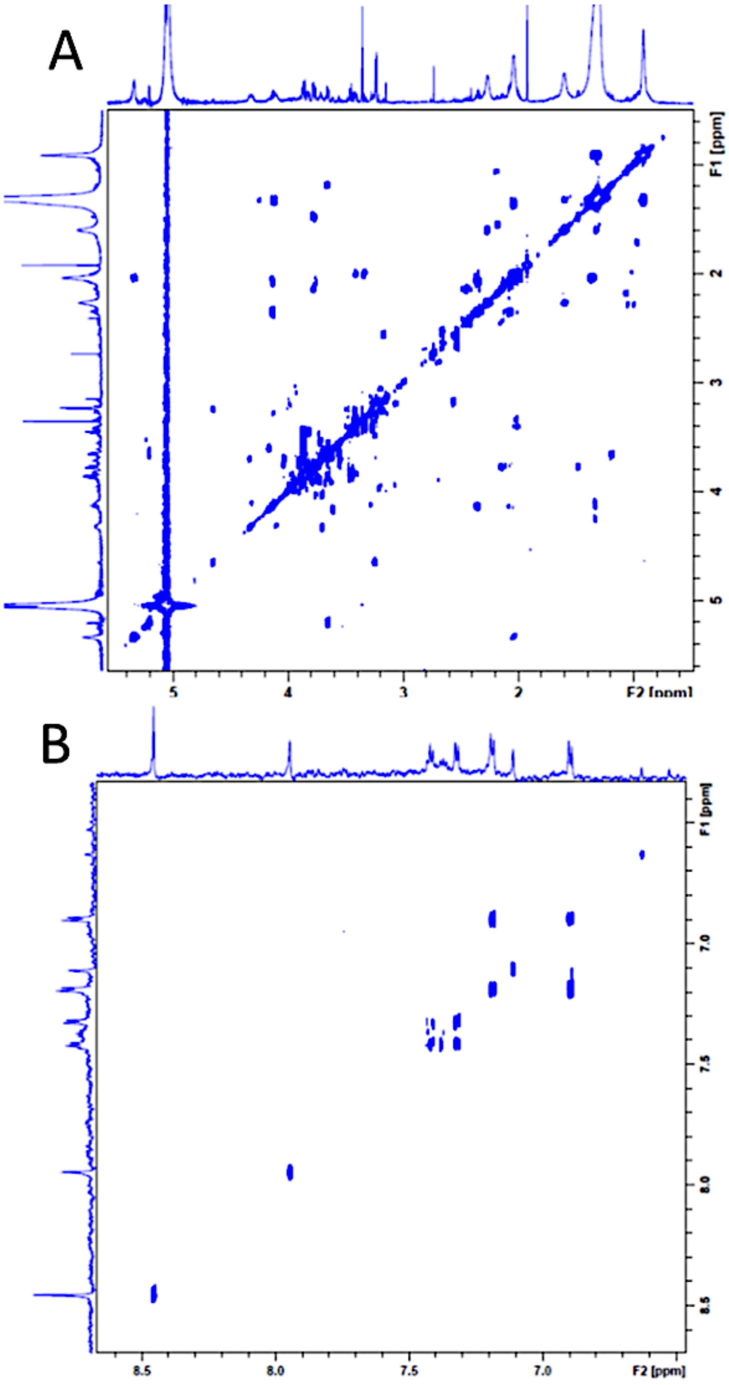


**Supplementary Figure 4. $^1H$-$^1H$ COSY of untreated pooled gut polar extracts (N=5).**

COSY map (between 0.6-5.6 ppm (A) and 5.9-8.6 (B)) shows the correlations between the protons of a metabolite which are coupled with each other indicating a close proximity.

A presaturation for the suppression of the residual water signal. (A) The aliphatic region and (B) the aromatic area. The map was recorded in 6 hours with 4096 x 388 data points, then zero-filled to 8k x 1k and apodized by sine functions along both dimensions prior to 2D Fourier transform. The recycling delay was 2 s, the number of scans 32.

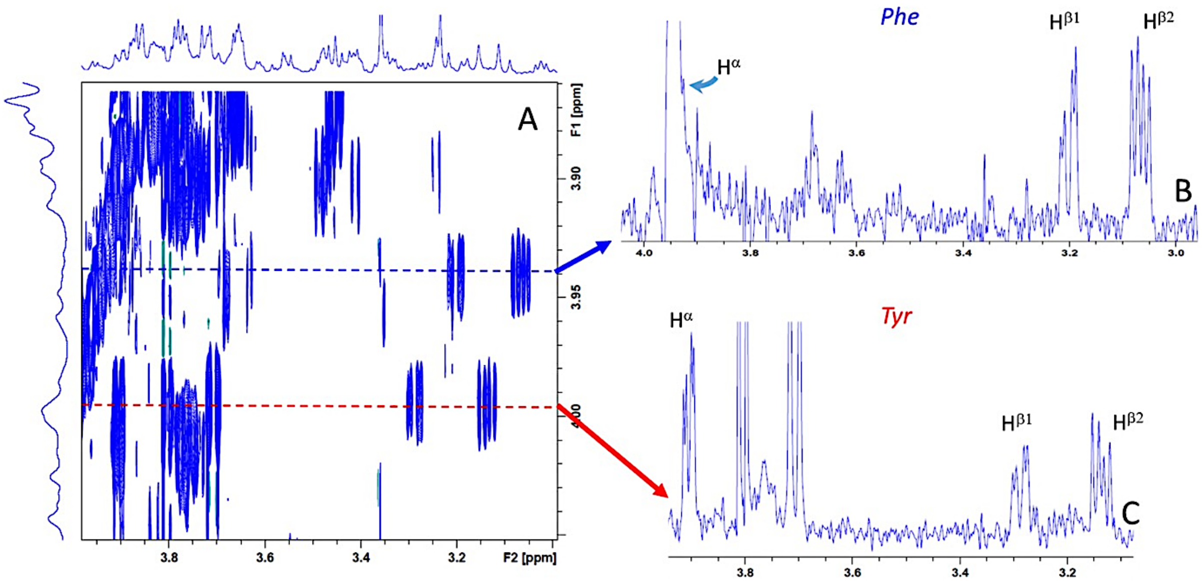


**Supplementary Figure 5. 2D ZQF TOCSY of untreated pooled gut polar extracts (N=5).**

The total correlation spectropy (TOCSY) shows all the correlations between protons in a molecule, not just between geminal or vicinal as in COSY.

(A) Zoom of the 2D ZQF TOCSY coming from the double Fourier transform of 6k x 500 points zero-filled to 8k x 1k and apodized with a cosine function along each dimension. Other relevant parameters are a recycling time d1 = 3 s, a mixing time of 150 ms and a number of accumulations NS = 32. (B) Extracted row corresponding to aliphatic signals of Phe. (C) Extracted row corresponding to aliphatic signals of Tyr.

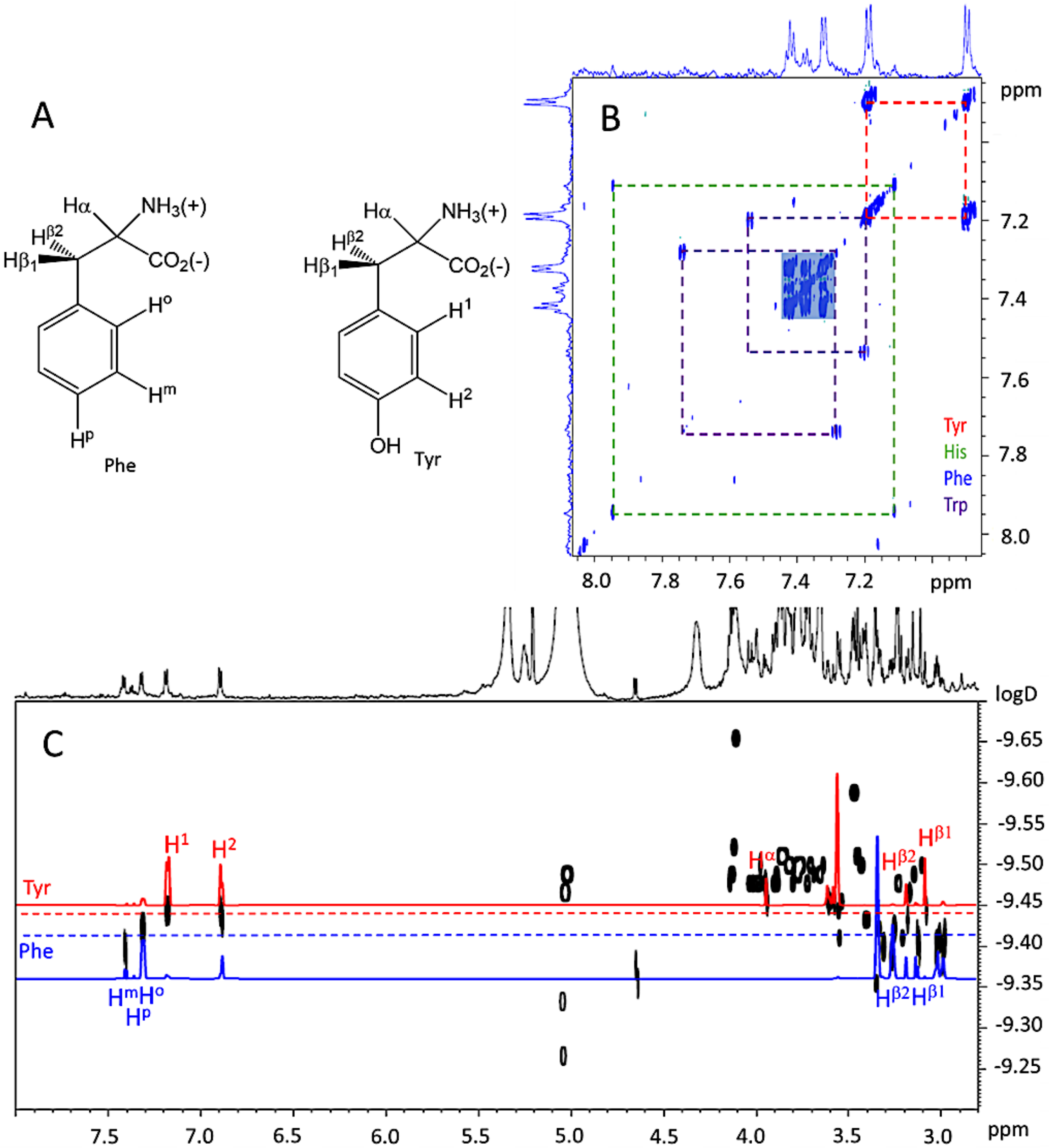


**Supplementary Figure 6. 2D ZQF TOCSY and 2D $^{1}$H DOSY spectra in the aromatic area.**

(A) Numbered structure of Phenylalanine (Phe) and Tyrosine (Tyr) (B) 2D ZQF TOCSY spectra of a gut extract from untreated ants (N=5) showing the aromatic amino acid signals of tyrosine, histidine, phenylalanine and tryptophan (C) 2D $^{1}$H DOSY spectra of gut extract for untreated ants showing the differential diffusion coefficient of Tyr and Phe. The red and blue lines are extracted lines of the 2D map showing all expected signals of the Tyr and Phe signals.

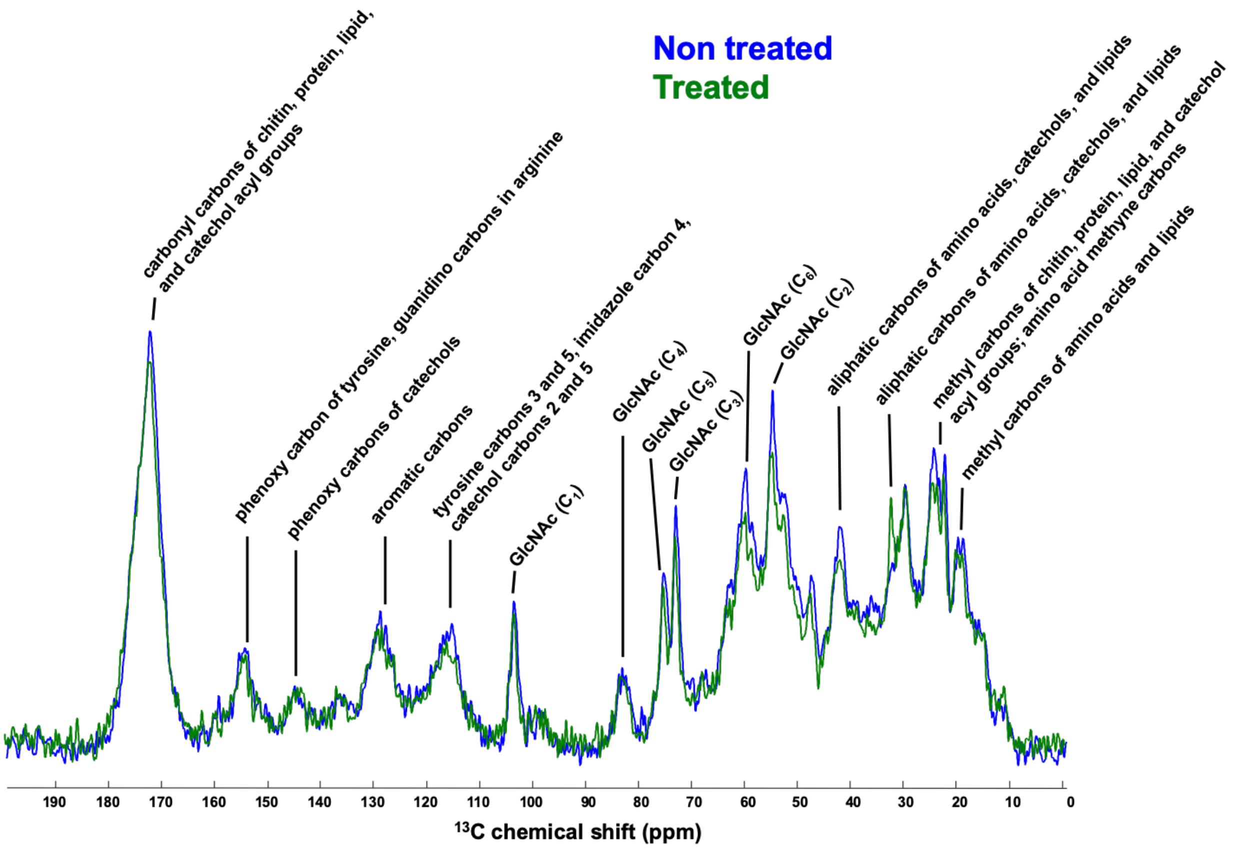


**Supplementary Figure 7. CP-MAS $^{13}$C NMR spectra of antibiotic treated and untreated samples.**

Both spectra of isolated cuticles are normalized with respect to sample mass in the rotor and the number of accumulations.

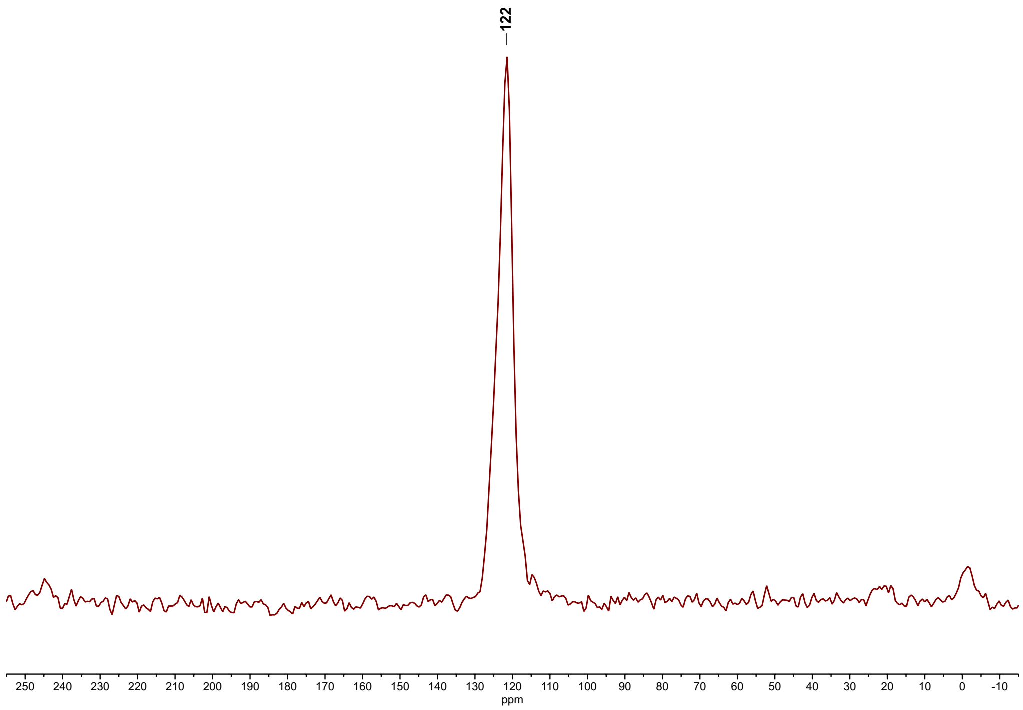


**Supplementary Figure 8. CP-MAS $^{15}N$ NMR spectra of α-chitine.**

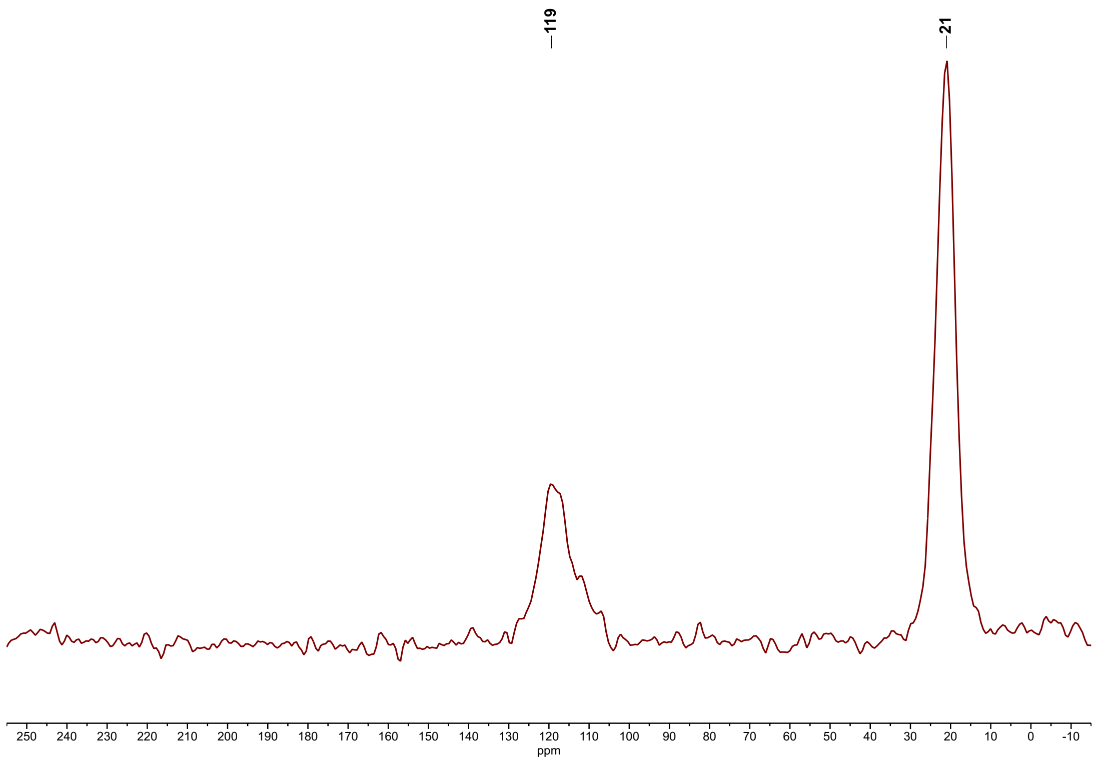


**Supplementary Figure 9. CP-MAS $^{15}N$ NMR spectra of chitosan (85% purity).**

The peak at 21 ppm represents the signal of amine in chitosan and the peaks at 119 ppm corresponds to the amide signal in chitin.

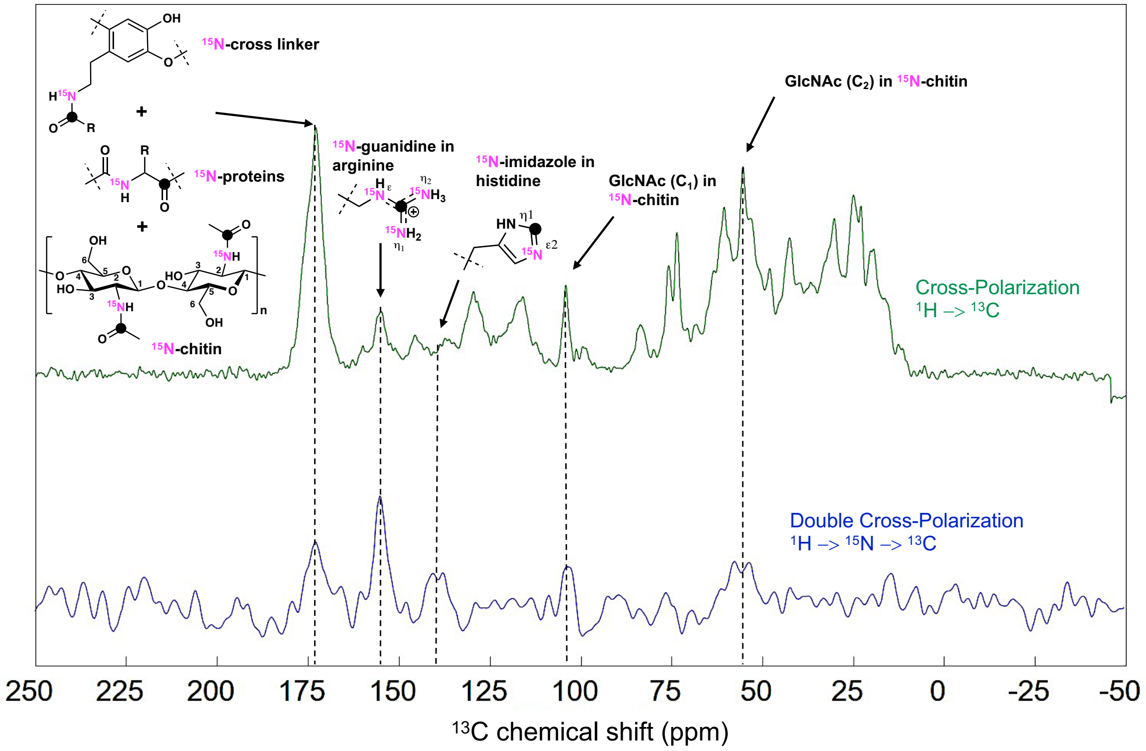


**Supplementary Figure 10. Double CP-MAS $^{13}C$ NMR spectra of the cuticle of $^{15}N$ labelled untreated *C. varians* individuals.**

The black dot on the chemical structure corresponds to the $^{13}C$ signal shown by the arrow.

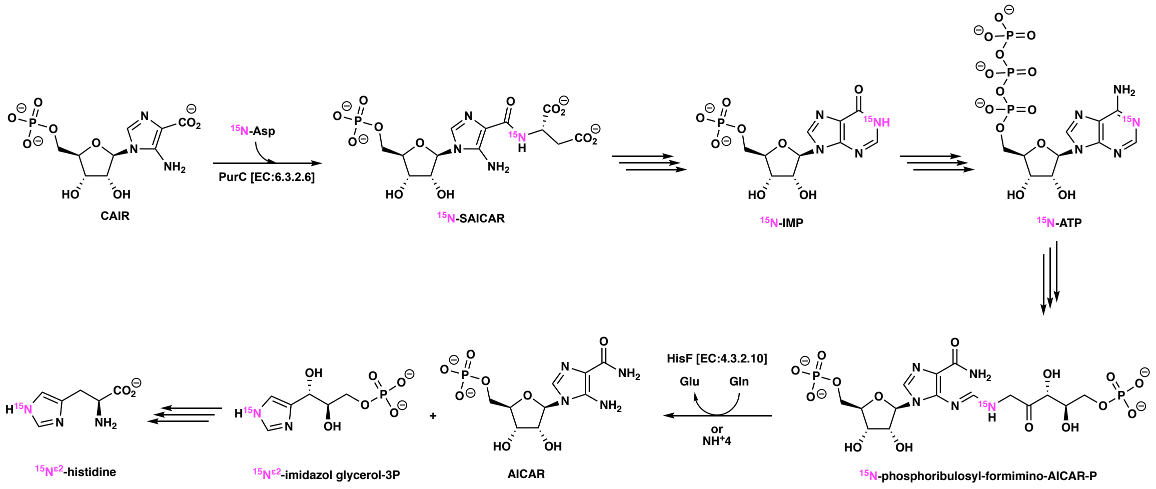


**Supplementary Figure 11. Putative pathway of the enriched $^{15}N^{\varepsilon 2}$-histidine biosynthesis in ants.**

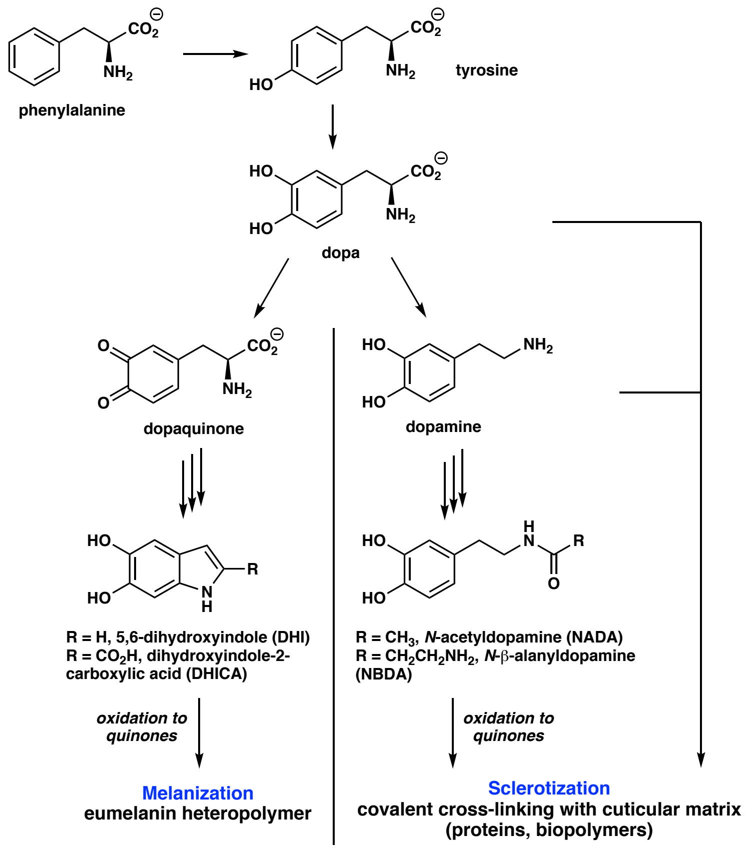


**Supplementary Figure 12. Biosynthetic pathways of sclerotization and melanization.**

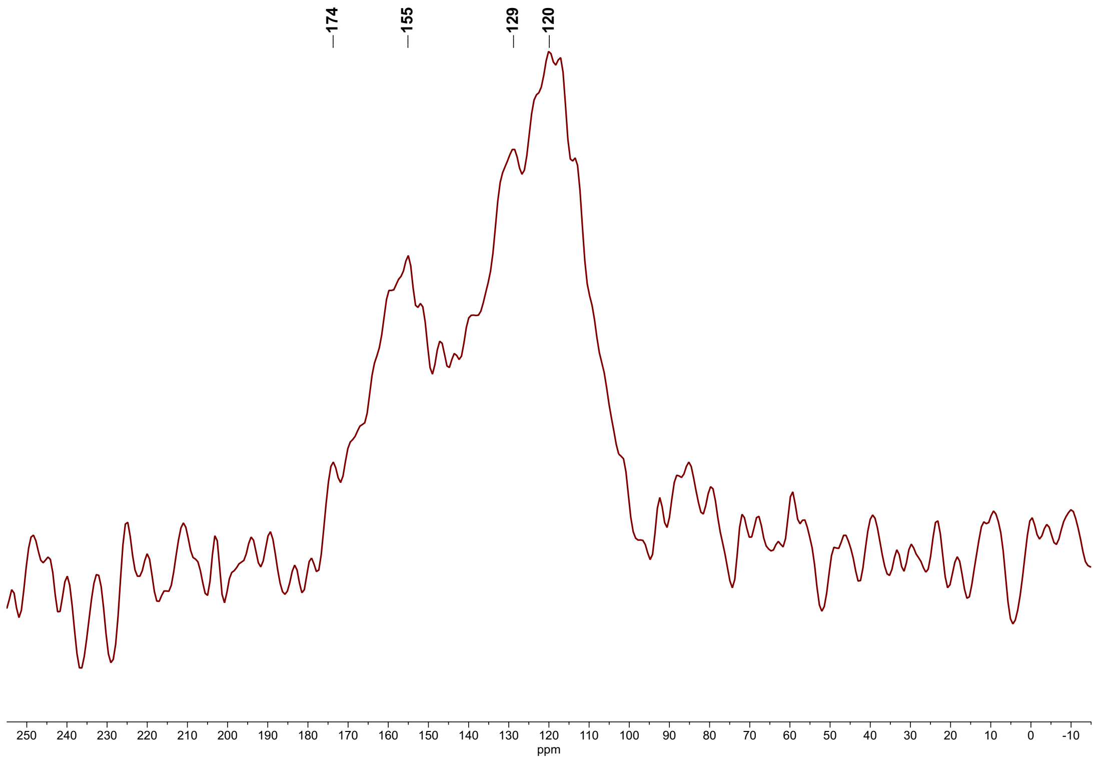


**Supplementary Figure 13. CP-MAS $^{15}$N NMR spectra of melanin from *Sepia officinalis.***

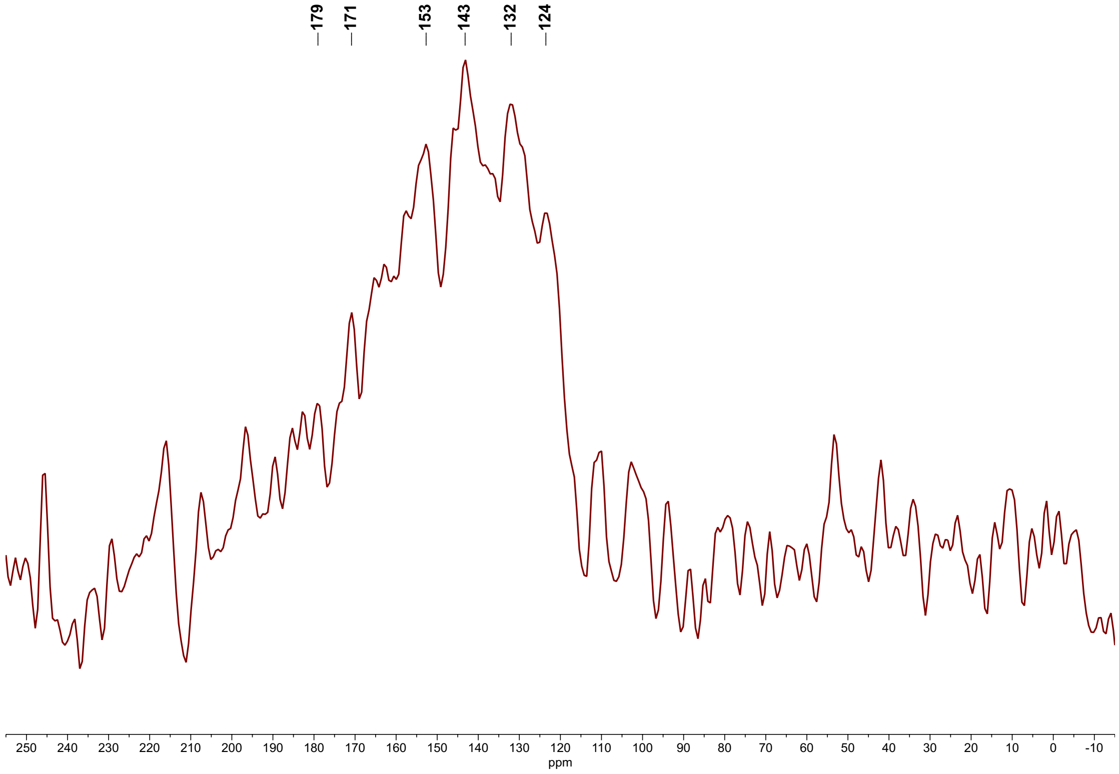


**Supplementary Figure 14. CP-MAS $^{15}N$ NMR spectra of synthetic melanin.**

**Supplementary Table 1. Bacterial quantification (qPCR) of the 16S rRNA gene (515F / 806R).**

| ***C. varians* samples** | **Cycle of quantification (Cq)** | **Sample copy number** | **Average** | **Error** |
|---|---|---|---|---|
| Untreated samples replicate 1 | 20.67 | 12,859.39 | | |
| Untreated samples replicate 2 | 20.70 | 12,603.95 | 14,033.07 | 2,257.70 |
| Untreated samples replicate 3 | 20.34 | 16,635.86 | | |
| Antibiotic treated replicate 1 | 21.66 | 5,978.97 | | |
| Antibiotic treated replicate 2 | 21.57 | 6,392.12 | 6,607.84 | 760.04 |
| Antibiotic treated replicate 3 | 21.38 | 7,452.43 | | |

**Supplementary Table 2. $\delta^{15}$N measurement in the cuticle by ICP-MS.**

| Sample ID | Weight (mg) | N2 Amp | %N | AT% 15N/14N | $\delta$15N vs. At. Air |
|---|---|---|---|---|---|
| C | 0.347 | 1,257 | 12.32 | 0.36 | 3.91 |
| TL | 0.228 | 812 | 13.41 | 4.73 | 12,509.03 |
| NTL | 0.379 | 1,011 | 12.65 | 15.83 | 50,172.15 |

C: control sample with no antibiotics and no labelled $^{15}$N-urea

TL: antibiotics treated labelled $^{15}$N-urea samples

NTL: antibiotics non-treated labelled $^{15}$N-urea samples

N2 Amp: the amplitude of the sample peak in mV of the respective gaz

Delta value are measured in units of per mil (‰)

$\delta$15N vs. At. Air: Corrected isotope delta value measured against a primary reference scale (atmospheric air)

**Supplementary Table 3. 1H NMR quantification of aromatic amino acids.**

| *C. varians* samples | Tyrosine concentration in µM (RMSD) | Phenylalanine concentration in µM (RMSD) |
|---|---|---|
| Untreated sample | 11.3 (0.1) | 11.2 (0.3) |
| Antibiotic treated sample | 4.9 (0.3) | 4.7 (0.3) |

Each sample: n=1 (pool of 5 workers)

Four experiments per sample, 2h per sample

RMSD: root-mean square deviation

**Supplementary Table 4. Chemical assignments of resonances in the CP-MAS $^{15}N$ NMR spectra of *Cephalotes varians* cuticle.**

| δ value (ppm) | Attribution | Assignment |
|---|---|---|
| 153 | 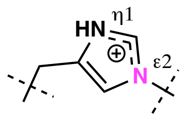 | N-substituted nitrogen ($N^{\varepsilon 2}$)<br>in protonated histidine |
| 139 | 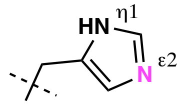 | nitrogen ($N^{\varepsilon 2}$)<br>in neutral histidine (tautomer II) |
| 145-100 | 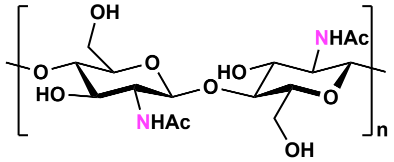<br>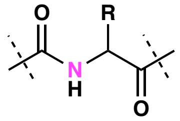<br>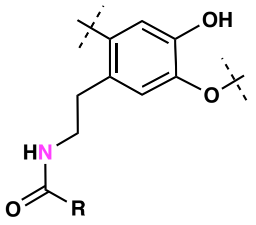 | amide in chitin, protein<br>and cross-linker |
| 95<br>77 | 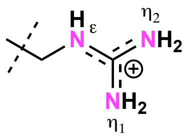 | nitrogen (Nε) in guanidine<br>nitrogen (Nη) in guanidine |
| 45-40 | 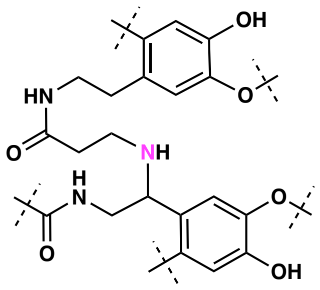 | intercatecholamide cross-link |

Nitrogens denoted in pink correspond to the assigned δ value signals.
