# Supplementary material for: Gut bacteria are essential for normal cuticle development in herbivorous turtle ants
Source: Nat Commun. 2021 Jan 29;12:676. doi: 10.1038/s41467-021-21065-y (PMC7846594; doi:10.1038/s41467-021-21065-y)
Supplement: Supplementary file 2 — Reporting Summary [file 41467_2021_21065_MOESM2_ESM.pdf]

nature research

Corresponding author(s): Christophe Duplais and Corrie Moreau

Last updated by author(s): Aug 13, 2020

# Reporting Summary

Nature Research wishes to improve the reproducibility of the work that we publish. This form provides structure for consistency and transparency in reporting. For further information on Nature Research policies, see our Editorial Policies and the Editorial Policy Checklist.

## Statistics

For all statistical analyses, confirm that the following items are present in the figure legend, table legend, main text, or Methods section.

| n/a | Confirmed | |
|---|---|---|
| ☐ | ☒ | The exact sample size (*n*) for each experimental group/condition, given as a discrete number and unit of measurement |
| ☐ | ☒ | A statement on whether measurements were taken from distinct samples or whether the same sample was measured repeatedly |
| ☒ | ☐ | The statistical test(s) used AND whether they are one- or two-sided<br>*Only common tests should be described solely by name; describe more complex techniques in the Methods section.* |
| ☒ | ☐ | A description of all covariates tested |
| ☒ | ☐ | A description of any assumptions or corrections, such as tests of normality and adjustment for multiple comparisons |
| ☒ | ☐ | A full description of the statistical parameters including central tendency (e.g. means) or other basic estimates (e.g. regression coefficient) AND variation (e.g. standard deviation) or associated estimates of uncertainty (e.g. confidence intervals) |
| ☒ | ☐ | For null hypothesis testing, the test statistic (e.g. *F*, *t*, *r*) with confidence intervals, effect sizes, degrees of freedom and *P* value noted<br>*Give P values as exact values whenever suitable.* |
| ☒ | ☐ | For Bayesian analysis, information on the choice of priors and Markov chain Monte Carlo settings |
| ☒ | ☐ | For hierarchical and complex designs, identification of the appropriate level for tests and full reporting of outcomes |
| ☒ | ☐ | Estimates of effect sizes (e.g. Cohen's *d*, Pearson's *r*), indicating how they were calculated |

*Our web collection on statistics for biologists contains articles on many of the points above.*

## Software and code

Policy information about availability of computer code

| | |
|---|---|
| Data collection | No software was used for data collection |
| Data analysis | QIIME 2.0 for bacterial community analyses; Bruker TopSpin for NMR spectra analysis |

For manuscripts utilizing custom algorithms or software that are central to the research but not yet described in published literature, software must be made available to editors and reviewers. We strongly encourage code deposition in a community repository (e.g. GitHub). See the Nature Research guidelines for submitting code & software for further information.

## Data

Policy information about availability of data

All manuscripts must include a data availability statement. This statement should provide the following information, where applicable:

- Accession codes, unique identifiers, or web links for publicly available datasets
- A list of figures that have associated raw data
- A description of any restrictions on data availability

Authors can confirm that all relevant data are included in the paper and/or its supplementary information files. Sequence data and NMR spectroscopic data are available respectively in the NCBI SRA database under the accession number PRJNA683914 and in the Dryad Digital Repository (https://datad ryad.org/) under https://doi.org/10.5061/dryad.d7wm37q0h.

# Field-specific reporting

Please select the one below that is the best fit for your research. If you are not sure, read the appropriate sections before making your selection.

☐ Life sciences ☐ Behavioural & social sciences ☒ Ecological, evolutionary & environmental sciences

For a reference copy of the document with all sections, see nature.com/documents/nr-reporting-summary-flat.pdf

# Ecological, evolutionary & environmental sciences study design

All studies must disclose on these points even when the disclosure is negative.

| | |
|---|---|
| Study description | This study has identified the cuticular components in ant cuticle which are enriched in nitrogen by gut bacteria highlighting the role of symbionts in insect evolution. We use gut bacterial manipulation, 15N isotopic enrichment, isotope-ratio mass spectrometry, and 15N nuclear magnetic resonance spectroscopy to gain insights into nitrogen assimilation in the ant cuticle. |
| Research sample | Cephalotes varians, the Florida turtle ant was selected as our previous research demonstrated this species use amino acids synthesized by their mutualistic gut bacteria (Hu et al. 2018 Nat Comm); all female life stages were included in the experiments. Control and antibiotic treated individuals were included. |
| Sampling strategy | Sample sizes were driven by the mortality of the live animals. We included all colonies that made it through the complete antibiotic treatment. Cuticle and intestinal tissues were carefully dissected under a compound microscope. |
| Data collection | The collect of ants, the feeding experiment and the 16S rRNA were perfomed by the Moreau Lab. Liquid NMR analysis of dissected ant guts was done by Martineau, Farjon and Giraudeau. Sarou-Kanian, Massiot, Hassan, Perrone conducted the solid-state NMR analysis of dissected cuticles. All NMR spectra were analyzed and interpreted by Duplais. Isotope ratio MS of dissected cuticles were done by Cornell Stable Isotope Laboratory. Estevez generated the electronic microscope images of dissected cuticles. |
| Timing and spatial scale | All data collection, across platforms, was gathered in a single event to limit bias of machine variation or errors. The antibiotic experiments were conducted June 15-July 7, 2017. All turtle ant samples are from a single population from the Florida Keys. |
| Data exclusions | No data were excluded from the analysis. |
| Reproducibility | As material was not sufficient to replicate experiments the major insight from this study is of qualitative nature. Using pooled material from multiple individuals is legitimate. |
| Randomization | Samples from independent colonies were combine to reduce bias from potential outliers. Splitting colonies across treatments allow us to control for genetic background and combining independent colonies for analysis insure colony specific variables are present across treatments. |
| Blinding | Analytical experiments were conducted without knowledge of treatment group to limit bias in data collection or intrepretation of results. |

Did the study involve field work? ☒ Yes ☐ No

## Field work, collection and transport

| | |
|---|---|
| Field conditions | Ant colonies were collected during spring 2017 in the subtropical mangrove forests in the Florida Keys. Colonies were collected during daylight hours on days without rain with an average daily temperature of 85-90°F. |
| Location | Cephalotes varians colonies (N=6) were collected in the Florida Keys, USA (24.691402, -81.189682) at sea level. |
| Access & import/export | Florida Department of Environmental Protection scientific research permit 04251635; U.S. Fish and Wildlife research permit FFO4RFKD-2015-0. |
| Disturbance | The disturbance was low as required by our collecting and research permits; population abudance of this species is very high in the Florida Keys but. Disturbance was minized by limiting the number of twig broken to collect ant colonies from mangrove trees. |

# Reporting for specific materials, systems and methods

We require information from authors about some types of materials, experimental systems and methods used in many studies. Here, indicate whether each material, system or method listed is relevant to your study. If you are not sure if a list item applies to your research, read the appropriate section before selecting a response.

## Materials & experimental systems

| n/a | Involved in the study |
|---|---|
| ☒ | ☐ Antibodies |
| ☒ | ☐ Eukaryotic cell lines |
| ☒ | ☐ Palaeontology and archaeology |
| ☐ | ☒ Animals and other organisms |
| ☒ | ☐ Human research participants |
| ☒ | ☐ Clinical data |
| ☒ | ☐ Dual use research of concern |

## Methods

| n/a | Involved in the study |
|---|---|
| ☒ | ☐ ChIP-seq |
| ☒ | ☐ Flow cytometry |
| ☒ | ☐ MRI-based neuroimaging |

# Animals and other organisms

Policy information about studies involving animals; ARRIVE guidelines recommended for reporting animal research

| | |
|---|---|
| Laboratory animals | This study did not involve laboratory animals |
| Wild animals | Wild colonies of the Florida turtle ant, Cephalotes varians, were collected in the field and transported back to the laboratory in styrofoam containers to minimize heat change exposure. All life stages were collected (eggs, larvae, pupae, workers, queens), but males were not present due to time of year. Animals were killed by being placed in a -80C freezer before dissections and analysis. |
| Field-collected samples | Live colonies were maintained in a standardized 12 hour photoperiod and temperature of 75°F and humidity were held constant for all colonies. |
| Ethics oversight | Only collecting permits were required for this project as ants/insects do not require IACAC approval. |

Note that full information on the approval of the study protocol must also be provided in the manuscript.
